# A Randomized Controlled Trial Comparing the Efficacy of Bilateral Percutaneous Tibial Nerve Stimulation Versus Biofeedback Pelvic Floor Muscle Training in the Management of Obstructed Defecation Syndrome

**DOI:** 10.7759/cureus.80885

**Published:** 2025-03-20

**Authors:** Fouad Ashoush, Ahmed Abdelrahim, Omer Ali, Dariush Kamali, Sanjay Harrison, Anil Reddy, Walid Elshazly, Mohamed Sultan, Tamer Saafan, Sabry Abounozha, Mooyad Ahmed

**Affiliations:** 1 General Surgery, Northumbria Healthcare NHS Foundation Trust, North Tyneside, GBR; 2 General Surgery, Health Education England North East, Newcastle, GBR; 3 Surgery, St. Mary's Hospital, Isle of Whight, GBR; 4 General Surgery, Darlington Memorial Hospital, Darlington, GBR; 5 General Surgery, James Cook University Hospital, Middlesbrough, GBR; 6 General Surgery, Alexandria Main University Hospital, Alexandria, EGY; 7 General Surgery, Cumberland Infirmary, Carlisle, GBR; 8 General Surgery, Sunderland Royal Hospital, Sunderland, GBR; 9 General Surgery, Royal Blackburn Teaching Hospital, Blackburn, GBR

**Keywords:** biofeedback, constipation, electrostimulation therapy, muscle training, pelvic floor, randomized controlled trial, transcutaneous nerve stimulation, treatment outcome

## Abstract

Introduction

Obstructed defecation syndrome (ODS) is a common disorder in developed countries. This study aims to compare the efficacy of bilateral percutaneous tibial nerve stimulation (Bi-PTNS) to biofeedback therapy (BFT) in adult patients with ODS.

Methods

A prospective randomised control study was conducted on patients aged ≥18 years, diagnosed with ODS, who were referred to the Colorectal Surgery Department at a main university hospital between 2018 and 2020. Computerized 1:1 block randomization allocated patients into two groups: the bi-PTNS group and the BFT group. The Constipation Scoring System and Patient Assessment of Constipation Quality of Life Score (PAC-QoL) were used to assess the severity of the patient’s symptoms prior to and after treatment. The primary outcome was the improvement of the Constipation Scoring System. The secondary outcome was the PAC-QoL score.

Results

In total, 60 patients, with 38 females (mean of 43 years in the BFT group and 48 years in the Bi-PTNS group), were studied. Statistically significant differences were achieved in patients who underwent bi-PTNS compared to the BFT group (p < 0.001). The average improvement in the Constipation Scoring System score for the bi-PTNS group was 66.66% ± 8.44 compared to 47.36% ± 10.44 for the BFT group. The bi-PTNS group showed improvement in the PAC-QoL score (60.41% ± 4.03) compared with 42.59% ±6.25 in the BFT group.

Conclusion

The Bi-PTNS intervention was more effective than BFT in alleviating symptoms of OD compared to BFT, evidenced by improvements in both the Constipation Scoring System and PAC-QoL scores.

## Introduction

Normal defecation is an intricate process contingent upon the precise interplay between the somatic and autonomic nervous systems, involving the integration of sensory information, pelvic floor muscles and anal sphincters [1,2,3]. Obstructed defecation syndrome (ODS) is a prevalent disorder observed in clinical practice, which affects 3-18% of the adult population [4]. 

Patients with ODS usually experience difficult defecation following an urge to defecate followed by an attempt of evacuation that usually results in a feeling of incomplete evacuation of the rectum and the necessity for rectal and/or vaginal digitation or pressing on the perineum to achieve complete evacuation in 25% or more of their defecation attempts [5]. ODS is categorised into functional and mechanical subtypes. Functional ODS involves anismus (pelvic floor dyssynergia), idiopathic megarectum and descending perineum syndrome. Mechanical causes of ODS include rectocele, enterocele, intussusception, sigmoidocoel, rectal intussusception and external rectal prolapse [6,7].

Evaluation of patients with ODS involves a comprehensive history, clinical examination and various investigations such as colonoscopy or sigmoidoscopy, trans-anal or vaginal ultrasound (US), plain defecation proctoram or magnetic resonance defecography, anorectal manometry and the balloon expulsion test, dynamic perineal ultrasound and, lastly, pudendal nerve motor latency test, which is not commonly used in clinical practice. Other investigations may be utilized to rule out other causes of functional constipation, such as colonic transit studies [8-13].

Non-operative measures are the first line of management in patients with functional ODS. These include lifestyle modifications, bowel habit training and the use of laxatives, in addition to psychological counselling if needed. Biofeedback training (BFT) is commonly used in practice in patients with functional ODS, with a reported success rate of up to 80% [14-21]. If a mechanical cause is identified, surgical intervention is indicated to restore normal anatomy. Managing patients with functional ODS who fail to respond to BFT is challenging. Surgical intervention in this group of patients cannot be easily justified. This dilemma led to exploring different treatment modalities such as bilateral percutaneous tibial nerve stimulation (Bi-PTNS). Bi-PTNS has been commonly used in the treatment of functional pelvic floor disorders such as overactive bladder and faecal and urinary incontinence [14].

This article was previously posted to the Research Square preprint server on January 19, 2024 (DOI 10.21203/rs.3.rs-3864132/v1).

## Materials and methods

Patients and methods

**Figure 1:**
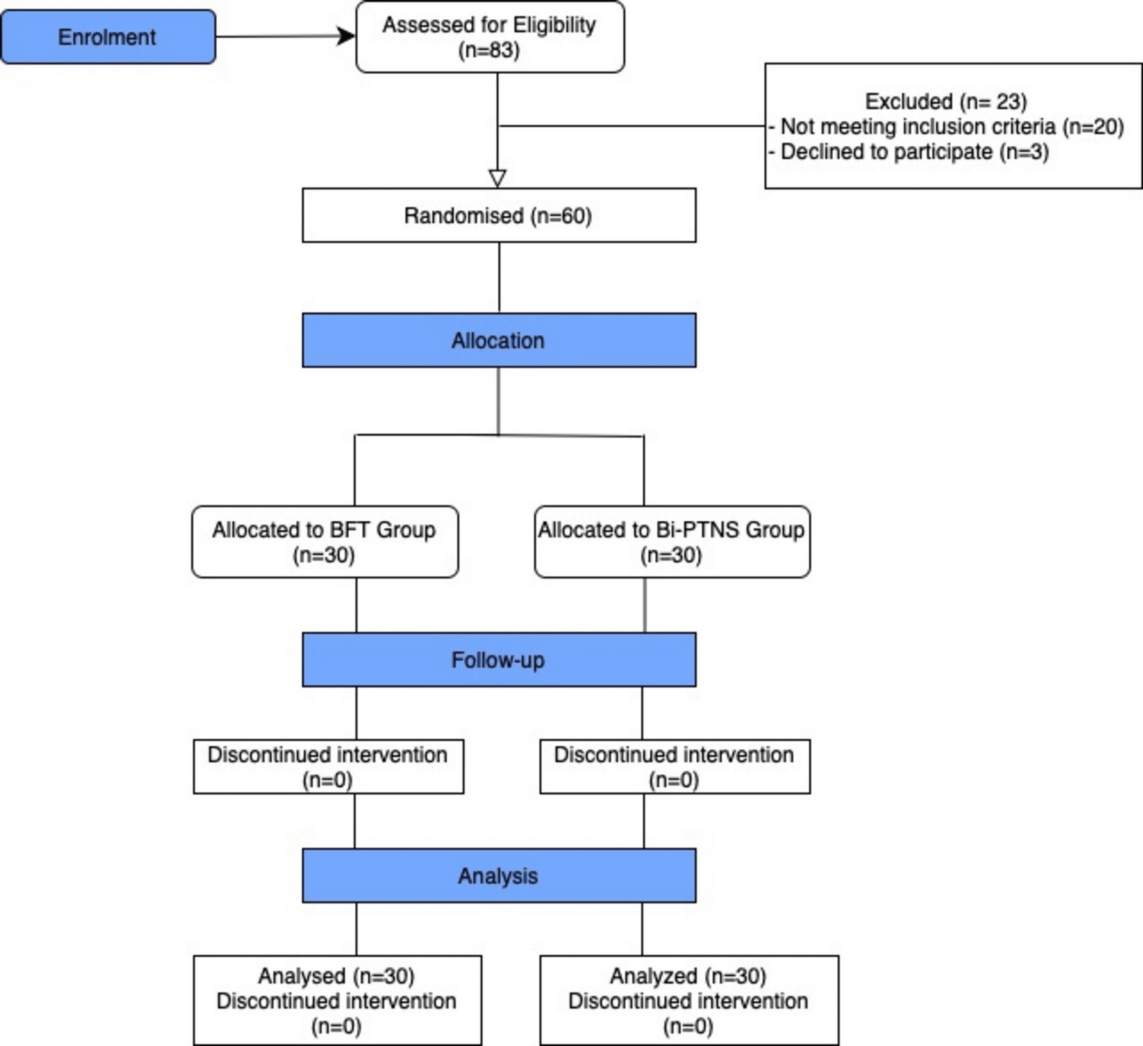
CONSORT flow diagram

A prospective randomized clinical trial was conducted at one of the main university hospitals between May 2018 and April 2022 on patients with ODS. Ethical approval was obtained from the local ethics committee of the Faculty of Medicine of Alexandria University in Alexandria, Egypt (serial number: 0106397). A written consent was secured from all participants. The study protocol was registered in the university post-graduate research registry. The study adhered to CONSORT reporting guidelines (Figure 1) [15]. The study was registered at the pan African Clinical Trial Registry (registration number: PACTR202008674682771). The study aims to compare the effectiveness of Bi-PTNS and BFT in the treatment of ODS.

The primary outcome measure was the improvement in the Constipation Scoring System [16]. The secondary outcome measure was the Patient Assessment of Constipation Quality of Life (PAC-QoL) score [17].

The study enrolled a sample of 60 eligible patients who were randomly assigned to either the BFT group (n = 30) or the Bi-PTNS (n = 30) using computerized randomization. Allocation concealment was rigorously implemented to prevent selection bias and ensure the integrity of randomization by utilising computer-generated random numbers by an independent statistician who had no involvement in participant recruitment or data collection. Patients were eligible for inclusion if they met the clinical diagnosis of ODS based on the ROME IV criteria [18]. This diagnosis required the presence of at least two of the following symptoms in ≥25% of defecation attempts for a minimum of three months: straining, lumpy or hard stools, sense of incomplete evacuation, sensation of anorectal obstruction/blockage or manual manoeuvres to facilitate defecation. Participants who fulfilled these clinical criteria underwent further investigation using imaging (defecography) and anal physiology studies. All eligible patients received initial conservative management, including dietary and lifestyle modifications, laxatives, bowel habit training and psychological counselling for a minimum of six weeks. The study excluded patients with evidence of structural causes of ODS, such as rectocele, enterocele and rectal prolapse. Individuals who demonstrated improvement following initial conservative measures were also excluded. Additional exclusion criteria encompassed: previous pelvic surgery or trauma; history of pelvic irradiation; known neurogenic causes including multiple sclerosis, Parkinson's disease and Hirschsprung disease; contraindications to Bi-PTNS (e.g., neuropathy, permanent pacemaker and soft tissue conditions affecting the site of electrode application); mental health problems precluding engagement in biofeedback therapy, metabolic disorders (including chronic hypokalemia, hypercalcemia, hypothyroidism and chronic kidney diseases); rheumatoid disorders; inflammatory bowel diseases; slow transit constipation; and anorectal congenital anomalies.

The sample size was determined through a power calculation aiming to achieve a statistical power of 80% to detect meaningful differences between the treatment groups. The calculation was based on relevant parameters extracted from previous studies, which served as a reference for effect size, significance level and anticipated variability [19].

The patients were subjected to thorough clinical evaluation by a blinded colorectal clinician. Clinical examinations, including assessment of perineal decent, perineal sensation, rest and squeezing anal sphincter muscle tone, were conducted. Female patients were assessed for the presence of rectovaginal fistula, rectovaginal septum weakness, rectocele, cystocele and uterine prolapse by means of clinical examination and imaging. Patients with suspected structural abnormalities were further investigated by dynamic magnetic resonance imaging (MR defecography). Images were obtained at rest, at maximum anal sphincter contraction, during straining, and during defecation/evacuation in the midsagittal plane. Patients with suspected anismus were subjected to nerve conduction studies and electromyography (EMG). At the initial assessment, all patients received education and illustrations about defecation physiology and anatomy of the pelvic floor. They were also educated about diet, bowel habits and defecation behaviours. All patients included in the study had reported failure of the conservative and lifestyle-changing measures for at least six weeks. Pre-treatment Constipation Scoring System and PAC-QoL scores were recorded.

Patients were assigned to one of the study groups by an independent, blinded researcher. In the BFT group, rectal and abdominal sensors were used to detect pelvic floor muscle signals, with 10 to 12 sessions in total, three sessions weekly lasting 30 minutes each, patients were asked to contract and relax their pelvic floor muscles guided by audiovisual response recorded on the BFT machine. Sessions were continued for 30 minutes unless the patient reported pain or asked to stop. For Bi-PTNS, transcutaneous Bi-PTNS was performed for 30 minutes duration unless the patient reported pain or asked to stop the session, three times per week. An average of 10 to 12 sessions were given. Transcutaneous stimulation was achieved by surface adhesive electrodes placed at the site of the tibial nerve with a negative electrode put behind the medial malleolus and a positive one 10 cm above it. All treatment sessions were performed by the same practitioner, who has had over 10 years of experience in pelvic floor physiotherapy.

The two groups of patients were all treated by the same physicians and evaluated at the end of the treatment period. All research subjects received the same standard medication, treatment strategies and necessary behavioural education before BFT or Bi-PTNS. An independent researcher collected the data on the post-treatment efficiency of each group using the Constipation Scoring System and PAC-QoL scores. Patients were assessed immediately after completing the assigned treatment course and 12 weeks later. The average scores were utilized for analysis. None of the patients were lost to follow-up.

Statistical analysis of the acquired data was conducted using IBM SPSS Statistics for Windows, Version 20.0 (released 2011, IBM Corp., Armonk, NY) [20]. Qualitative data were described using numbers and percentages. The Kolmogorov-Smirnov test was employed to ascertain the normality of distribution. Quantitative data were described using range (minimum and maximum), mean and standard deviation or median and interquartile range (IQR). The statistical significance of the obtained results was set at the 5% level. A chi-square test was used for categorical variables to compare between different groups. Categorial variables were subjected to the Chi-square test, with Fisher’s exact or Monte Carlo correction applied when more than 20% of cells exhibited expected counts below 5. For normally distributed quantitative variables, the Student t-test was applied for intergroup comparisons, and the paired t-test was used for intergroup analysis across different periods.

## Results

No statistically considerable differences between both groups in the demographic data including age, sex, parity and occupation were found. Twenty-nine patients were excluded. Most of the patients were excluded due to the presence of structural pelvic floor pathology, e.g., rectal prolapse and rectocele. Two patients had previous pelvic radiation, and one patient had previous severe pelvic trauma. Out of 60 patients included in the study, 22 were males and 38 were females. The mean age for the BFT group was 43.63 ± 14.32 years, while in the Bi-PTNS group, the mean age was 48.13 ± 10.71 years with no statistically significant variance (Table 1).

**Table 1:** Comparison between the two studied groups according to demographic data n: number of individuals. Group I: biofeedback therapy (BFT) group; Group II: bilateral percutaneous tibial nerve stimulation (Bi-PTNS) group

-	Group I (n = 30)	Group II (n = 30)
-	N (%)	N (%)
Sex	-	
Male	10 (33.3)	12 (40.0)
Female	20 (66.7)	18 (60.0)
Age (years)	-	
<30	4 (13.8)	2 (6.7)
30 – <40	10 (34.5)	3 (10.0)
40 – <50	5 (17.2)	8 (26.7)
50 – <60	8 (27.6)	15 (50.0)
≥60	2 (6.9)	2 (6.7)
Parity	-	
Nulliparous	4 (20.0)	4 (22.2)
Multiparous	16 (80.0)	14 (77.8)
Occupation	-	
Not working	8 (26.7)	5 (16.7)
Housewife	12 (40.0)	8 (26.7)
Office work	5 (16.7)	12 (40.0)
Manual work	5 (16.7)	5 (16.7)

There were no statistical differences between both groups in the presence of perineal descent (Table 2) and the presence of anismus (Table 3). 

**Table 2:** Comparison between the two studied groups according to perineal descent χ2: Chi-square test; p: p-value. Group I: biofeedback therapy (BFT) group; Group II: bilateral percutaneous tibial nerve stimulation (Bi-PTNS) group

Clinical examinations	Group I (n = 30)	Group II (n = 30)	^x2^	p
	N (%)	N (%)		
Perineal descent	-			
Present	11 (36.7)	12 (40.0)	0.071	0.791
Absent	19 (63.3)	18 (60.0)		

**Table 3:** Comparison between the two studied groups according to the presence of anismus. χ2: Chi-square test; p: p-value. Group I: BFT group; Group II: Bi-PTNS group

Presence of anismus	Group I (n = 30) N (%)	Group II (n = 30) N (%)	χ^2^	p
Present	13 (43.3)	12 (40.0)	0.069	0.793
Absent	17 (56.7)	18 (60.0)		

Although there was no significant difference between both groups in the baseline Constipation Scoring System, post-treatment analysis demonstrated a statistically significant improvement in the constipation score for the Bi-PTNS group. The average improvement in the Constipation Scoring System score for the Bi-PTNS group was 66.66% ± 8.44 as compared to 47.36% ± 10.44 for the BFT group (Table 4). Similarly, there was no statistically significant difference between both groups in the baseline PAC-QoL score. Following the intervention, patients subjected to Bi-PTNS exhibited a significant improvement in their score (60.41% ± 4.03) compared with 42.59% ± 6.25 in patients who received BFT (Table 5).

**Table 4:** Comparison between the two studied groups according to Constipation Scoring System pre and post treatment. t: Student t-test; IQR: interquartile range. Group I: biofeedback therapy (BFT) group; Group II: bilateral percutaneous tibial nerve stimulation (Bi-PTNS) group

Constipation Scoring System score	Group I (n = 30)	Group II (n = 30)	t	p-value
Pre-treatment	-	-	-	
Min. – Max.	11.0 – 19.0	10.0 – 20.0	0.112	0.911
Mean ± SD.	15.73 ± 1.91	15.67 ± 2.63		
Median (IQR)	16.0 (14.0 – 17.0)	16.0 (14.0 – 18.0)		
Post-treatment	-			
Min. – Max.	4.0 – 12.0	2.0 – 9.0	5.506^*^	<0.001^*^
Mean ± SD.	8.40 ± 2.36	5.33 ± 1.94		
Median (IQR)	8.0 (6.0 – 10.0)	5.0 (4.0 – 7.0)		
p-value	<0.001^*^	<0.001^*^	-	
% of decrease	47.36 ± 10.44	66.66 ± 8.44	7.872^*^	<0.001^*^

**Table 5:** Comparison between the two studied groups according to the Patient Assessment of Constipation Quality of Life (PAC-QoL) score. t: Student t-test; IQR: interquartile range; p: p-value for comparing between the studied groups. p1: p-value for paired t-test for comparing between the initial and post-treatment at 12 weeks *: Statistically significant at p ≤ 0.05 Group I:  BFT group - Group II: Bi-PTNS group

PAC-QoL score	Group I (n = 30)	Group II (n = 30)	t	p
Pre-treatment	-	-	-	-
Min. – Max.	54.0 – 81.0	55.0 – 83.0	0.156	0.876
Mean ± SD.	66.27 ± 6.31	66.53 ± 6.90		
Median (IQR)	66.0 (62.0 – 70.0)	66.0 (62.0 – 70.0)		
Post-treatment	-			
Min. – Max.	29.0 – 50.0	20.0 – 32.0	9.415^*^	<0.001^*^
Mean ± SD.	38.17 ± 6.35	26.23 ± 2.81		
Median (IQR)	37.50 (32.0 – 45.0)	27.0 (25.0 – 28.0)		
p_1_	<0.001^*^	<0.001^*^	-	
% of decrease	42.59 ± 6.25	60.41 ± 4.03	13.124^*^	<0.001^*^

## Discussion

ODS is a prevalent pelvic floor disorder, which poses a significant health burden in developed countries [21]. The multifaceted landscape of its management necessitates the exploration and refinement of therapeutic modalities. Following the inadequate response to the initial pharmacological treatment with laxatives, behavioural therapy and bowel habit training, BFT stands out as the preferred treatment option. Multiple randomized controlled studies have demonstrated that BFT resulted in a significant improvement of symptoms in patients with functional ODS, with effectiveness reaching up to 80% and a long-term success rate of nearly 50% compared to oral laxative therapy [22-23].

PTNS is an alternative therapeutic avenue that exhibits promising outcomes in the management of patients with ODS. It may be utilized as an alternative or adjunct to BFT. PTNS has been used in the treatment of various pelvic disorders such as overactive bladder and faecal incontinence [24-26]. The definite theory of how this achieved improvement with PTNS remains indistinct. It is hypothesized that PTNS acts by direct modification of the peripheral nerve roots, which have the same spinal roots as the nerves supplying the pelvic floor muscles (L4-S3), or by stimulating the cortical pontine centres, leading to the amplification of the somato-visceral reflexes, resulting in improvement in the pelvic floor muscle function [26].

Gocke et al. found that PTNS reduced the time patients spent in the toilet. It resulted in a statistically significant decrease in the use of softeners, obstructive symptoms and colonic inertia symptoms after the six-week treatment period. The treatment effect lasted for at least 12 weeks [27]. Velasco-Benitez et al. suggested that PTNS may achieve significant improvement in functional constipation in children [28]. Madbouly et al. concluded that PTNS resulted in a significant improvement of the modified obstructed defecation score (MODS) in patients with functional ODS [29]. A prospective randomized controlled trial by Pakghalb et al. found that PTNS resulted in significantly better results than BFT in patients with pelvic floor dyssynergia [30]. 

In our study, both treatment groups showed improvement in the Constipation Scoring System and PAC-QoL score, yet a more pronounced effect was observed in the Bi-PTNS group. In the BFT group, the Constipation Scoring System decreased from a mean of 16 to 8 with a 50% decrease after treatment, while in the Bi-PTNS group, the Constipation Scoring System decreased from a mean of 16 to 5 with a 66% decrease after treatment. Furthermore, the PAC-QoL echoed this trend. In the BFT group, the score diminished from a mean of 66 to 37, with a 42% decrease after treatment. While in the Bi-PTNS group, the PAC-QoL score diminished from a mean of 66 to 27, with a 60% decrease after treatment. The complex mechanism of action of Bi-PTNS may contribute to this discrepancy as it may contribute to its effectiveness in a certain group of patients with ODS.

With this study being a single-centre study, with a small number of patients and reporting only short-term outcomes after interventions, which may affect the generalizability of our data on patients in the rest of the world, longer-term data may be beneficial. The use of the Constipation Scoring System is standard practice for patients with ODS in our unit. We admit that other scores, such as the MODS, are more commonly used for patients with ODS. However, there is a significant overlap between the two scores.

## Conclusions

The results of this study suggest that using Bi-PTNS in patients with functional ODS is superior in controlling patient’s symptoms than BFT as evidenced by the significant improvements in the Constipation Scoring System and PAC-QoL scores. The minimally invasive and safe nature of Bi-PTNS positions it as a useful intervention for non-anatomic cases of ODS. These findings contribute valuable insights to pelvic floor disorder management, highlighting the efficacy of Bi-PTNS. However, further research is needed for a better understanding of the underlying mechanisms and to refine treatment protocols for optimal outcomes. This study lays the groundwork for future investigations aimed at advancing therapeutic strategies for individuals dealing with ODS.
